# Periodontal Disease and Other Adverse Health Outcomes Share Risk Factors, including Dietary Factors and Vitamin D Status

**DOI:** 10.3390/nu15122787

**Published:** 2023-06-17

**Authors:** William B. Grant, Barbara M. van Amerongen, Barbara J. Boucher

**Affiliations:** 1 Sunlight, Nutrition and Health Research Center, P.O. Box 641603, San Francisco, CA 94164-1603, USA; 2 Independent Researcher, Veemkade 466, 1019 HE Amsterdam, The Netherlands; bmvanamerongen@gmail.com; 3 The Blizard Institute, Barts and The London School of Medicine and Dentistry, Queen Mary University of London, London E1 2AT, UK; bboucher@doctors.org.uk

**Keywords:** asthma, cancer, cardiovascular disease, chronic obstructive pulmonary disease, COVID-19, Mendelian randomization, myocardial infarction, observational studies, pregnancy, randomized controlled trials

## Abstract

For nearly a century, researchers have associated periodontal disease (PD) with risks of other adverse health outcomes such as cardiovascular disease, diabetes mellitus, and respiratory diseases, as well as adverse pregnancy outcomes. Those findings have led to the hypothesis that PD causes those adverse health outcomes either by increasing systemic inflammation or by the action of periodontopathic bacteria. However, experiments largely failed to support that hypothesis. Instead, the association is casual, not causal, and is due to shared underlying modifiable risk factors, including smoking, diet, obesity, low levels of physical activity, and low vitamin D status. Diabetes mellitus is also considered a risk factor for PD, whereas red and processed meat are the most important dietary risk factors for diabetes. Because PD generally develops before other adverse health outcomes, a diagnosis of PD can alert patients that they could reduce the risk of adverse health outcomes with lifestyle changes. In addition, type 2 diabetes mellitus can often be reversed rapidly by adopting an anti-inflammatory, nonhyperinsulinemic diet that emphasizes healthful, whole plant-based foods. This review describes the evidence that proinflammatory and prohyperinsulinemia diets and low vitamin D status are important risk factors for PD and other adverse health outcomes. We also make recommendations regarding dietary patterns, food groups, and serum 25-hydroxyvitamin D concentrations. Oral health professionals should routinely inform patients with PD that they could reduce their risk of severe PD as well as the risks of many other adverse health outcomes by making appropriate lifestyle changes.

## 1. Introduction

The term periodontal disease (PD) has its origins in the Greek *peri* (around) and *odous* (tooth) [1]. It is a gum disease that starts as gingivitis, a reversible inflammation, which can develop into periodontitis, an irreversible inflammation. Gingivitis is an inflammatory disorder resulting from interactions between dental plaque biofilm and the host’s immune-inflammatory response, which remains contained within the gingiva and does not extend to the periodontal attachment (cementum, periodontal ligament, and alveolar bone). Being confined to the gingiva, such inflammation does not extend beyond the mucogingival junction and is reversible by reducing levels of dental plaque at and apical to the gingival margin [2]. Clinically, gingivitis is characterized by red, swollen, warm, painful gums with loss of function or simply bleeding gums. The inflammation is a response to a dental plaque biofilm—bacteria that have been allowed to accumulate on the teeth. The oral microbiome comprises not only bacteria but also viruses, fungi, archaea, and protozoa that live in the oral cavity [3]. The supra- or subgingival dental plaque biofilm can shift from a healthy symbiosis to a diseased dysbiotic biofilm [3,4]. Plaque may also mineralize, forming dental calculus that expands subgingivally along the root, stripping it and separating it from the alveolar bone. The shallow gingival sulci become deeper periodontal pockets. Once dental calculus is formed, it is difficult to remove.

**Figure 1:**
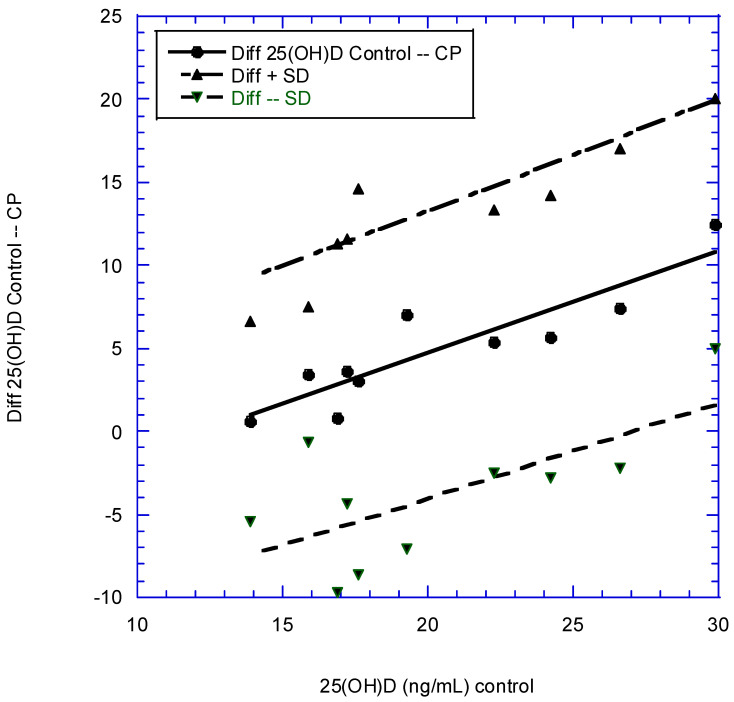
Plot of difference (Diff) and 95% confidence interval (CI) values in 25-hydroxyvitamin D [25(OH)D] concentrations between people with chronic periodontitis (CP) and matched controls, along with the linear regression fits to the data given in Table 8. Solid lines show regression fits to mean differences in serum 25(OH)D concentration, whereas dashed lines show regression fits to 95% CIs for differences. Regression fit to difference in 25(OH)D (ng/mL) = (7.6 + 0.61 × 25(OH)Dcontrol) ng/mL, *p* < 0.001. SD, standard deviation.

Periodontitis is a chronic multifactorial inflammatory disease associated with dysbiotic plaque biofilms and characterized by progressive destruction of the tooth-supporting tissues. Its main features include loss of periodontal tooth support, manifested through clinical attachment level loss (CAL) and radiographically assessed alveolar bone loss; the presence of periodontal pocketing; and gingival bleeding [5]. Usually, the gradual process takes place over years; the teeth start to loosen and can eventually be lost. The anatomical structure of the periodontium in health and periodontitis is shown in Figure 1 of a review published in 2019 [6]. Another review presents a schematic figure of the role of calculus in the etiology of periodontitis [7].

The gingival epithelial barrier is the first line of periodontal tissue defense against pathogenic bacteria. The breakdown of the “blood-tooth barrier,” often described as “leaky gums,” can lead to bacteremia, especially after chewing, thus allowing systemic host infection [8,9,10]. Low-grade inflammation is a recognized risk factor for several diseases, including cardiovascular disease (CVD), cerebrovascular neurodegenerative diseases, cancer, and PD [11]; thus, periodontitis may coexist with other systematic diseases.

According to data from the World Health Organization, the global prevalence of periodontitis (Community Periodontal Index of Treatment Needs code 3 + 4 [periodontal pocket 4–5 mm and 6+ mm]) varies from 10% in lower-middle income countries to 43% in upper-middle income countries [12]. In the United States, severe periodontitis in adults aged 30–70 years for 2009–2012, from National Health and Nutrition Examination Survey (NHANES) data, had a latitudinal gradient with estimates of 9.4–11.3% for southern states and 6.4–8.7% for northwestern states [13]. Those and similar datasets could be used in ecological studies to see what the correlations are with respect to such factors as ethnicity/race, obesity, poverty, and smoking, as has been conducted for cancer mortality rates [14]. 

The main risk factors for PD are thought to include smoking [15], diet [16], obesity [17], and diabetes mellitus [18,19]. The risk factors for systemic diseases are similar to those for PD, especially smoking [20,21], diet [22], obesity [23,24], and diabetes mellitus [25,26,27,28]. Low physical activity is also associated with an increased risk of PD [29]. 

This narrative review strives to determine how to interpret the evidence that people with PD have a significantly higher risk of developing other adverse health outcomes and then use that interpretation to make recommendations to improve their health. The steps used in reaching that goal included the following:

1. Review the history of associations between periodontitis or PD and other adverse health outcomes and determine whether PD is a causal risk factor for those adverse health outcomes, such as CVD, diabetes, and respiratory diseases;
2. Review the role of dietary risk factors for PD and other adverse health outcomes;
3. Review the evidence that vitamin D reduces the risk of PD as well as other adverse health outcomes;
4. Make recommendations regarding changes in dietary patterns and food groups and in serum 25-hydroxyvitamin D [25(OH)D] concentrations for reducing the risks of PD and other adverse health outcomes.

Using the National Library of Medicine’s PubMed database and Google Scholar, we searched the literature for the following terms: gingivitis, periodontitis, periodontal disease, diet, risk, meat, dietary patterns, vitamin D, 25-hydroxyvitamin D, Alzheimer’s disease, cancer, cardiovascular disease, dementia, diabetes, systemic diseases, chronic diseases, infectious diseases, respiratory diseases, pregnancy outcomes, recommendations, smoking, physical activity, inflammation, observational studies, randomized controlled trials, and Mendelian randomization. Papers were sought on the topics discussed, with preference for more recent publications, higher citation rates at Google Scholar, open access, and the quality of the research or review. Further, we sought reports that offered support and disagreement with the hypotheses considered.

## 2. Associations between Periodontal Disease and Other Adverse Health Outcomes

The finding of associations between PD and other adverse health outcomes has a long history. A 1983 review regarding diabetes and PD [30] reported 71 articles dating back to 1928 [31]. A 1952 article reported on the influence of systemic diseases on alveolar bone [32]. A 1994 review noted that people with many missing teeth had a high prevalence of coronary heart disease (CHD) and stroke [33]. The proposed reason for the link was smoking. The authors of a 1996 article hypothesized that the association between PD and the incidence of atherosclerosis and thromboembolic events was due to an underlying inflammatory condition leading to the development of both diseases [34]. A 2002 review noted that in nine cohort studies, chronic periodontitis (CP) was associated with a >15% increased risk of CHD, but that rigorous investigation would be required to determine whether the link was due to CP or lifestyle factors such as smoking [35]. A 2005 review stated that common forms of PD have been associated with adverse pregnancy outcomes, CVD, stroke, pulmonary disease, and diabetes, but that causal relations had not been established [36]. A 2009 review [37] pointed out that Cleave and Yudkin considered both dental caries and PD to be early warning signs of diabetes, obesity, and CHD. Because the onset of “[dental] decay [occurred] in (a) matter of months,” the onset of “diabetes maybe 20 years, coronary heart disease 30 years” [38], dental caries and pyorrhea (periodontitis) were considered alarm bells for future systemic diseases. Yudkin hypothesized that “people are laying the foundations for serious disease in later life by encouraging the development of a sweet tooth in children” [39].

By 2016, 57 systemic conditions had been hypothesized to be linked with PD, covering nearly 2% of the diseases indexed in MeSH [40]. A 2017 review noted that although the very nature of multifactorial chronic diseases makes it difficult to establish a definitive causal role for periodontal pathobionts in systemic infection, the body of literature supporting such an etiopathological role for those organisms is too substantial to be ignored as mere coincidence [41]. The review also said that well-controlled, large-scale prospective study designs or highly representative animal-model studies are needed to explore the relationship between a dysbiotic oral microbiome and systemic disease at a mechanistic level. A second 2017 article noted that periodontal bacteria or their antigens enter the circulatory system in relatively small amounts at any given time and are routinely eliminated by the immune system within minutes. That assertion makes arguing for a causative link between periodontal bacteria and a wide range of systemic diseases difficult, if not impossible. Moreover, bacterial pathogenicity has not been conclusively linked to the etiologic pathway of most common systemic diseases [42]. 

A 2018 article stated that during the past ~25 years, many researchers have reported associations between some oral diseases and conditions such as preterm birth, diabetes, CVD, stroke, and cancer. The point of those studies is that preventing and treating oral disease will modify, reduce, or prevent various systemic diseases. However, a need exists for more convincing and higher quality evidence that oral health care actually measurably affects specific systemic diseases before it can be claimed that attaining good oral health can prevent systemic diseases or conditions [43]. 

A 2019 review discusses periodontal medicine research conducted over the past 100 years, focusing on the effects of PD on three pathological conditions: CVD, diabetes mellitus, and adverse pregnancy outcomes. The authors selected 29 total studies that were the first of their kind in that they provided novel observations or contributed to shifting paradigms, as well as making strong contributions to progress in understanding the relationships of PD to systemic conditions. Overall, most cross-sectional, case-control, and longitudinal studies have revealed positive associations between poor periodontal status and CVD, diabetes metabolic control, and several adverse pregnancy outcomes, and those associations are upheld in systematic reviews. Findings from randomized controlled trials (RCTs) testing the effects of periodontal therapy on systemic health outcomes have been conflicting and inconsistent. Although a great deal of progress has been made, we highlight lessons learned and make comments and suggestions on several key aspects. Those aspects include the different definitions of PD cases used across studies, accounting for features of the periodontal phenotype most relevant to the biological link between periodontitis and systemic outcomes, the role of other comorbid inflammatory conditions, the selection of study participants, and the timing and intensity of periodontal intervention [44].

A 2019 review noted that a growing body of literature suggests a link between periodontitis and systemic diseases but that a causal relationship has not been established yet for most of the diseases and that the mediators of the association are still being identified [45].

A 2021 systematic review of reviews that studied how nonsurgical periodontal treatment (NSPT) affected systemic disease outcomes reported on the outcomes evaluated, categorizing them as biomarkers and as surrogate or clinical endpoints. In addition, those reviews used A MeaSurement Tool to Access Systematic Reviews 2 (AMSTAR 2) to evaluate the methodological quality of the reviews. Of the 52 studies included, 21 focused on diabetes, 15 on adverse birth outcomes, 8 on CVD, 3 each on obesity and rheumatoid arthritis, and 2 on chronic kidney disease. Across all studies, surrogate endpoints predominated as outcomes, followed by biomarkers and, rarely, actual disease endpoints. Ninety-two percent of studies had “low” or “critically low” AMSTAR 2 confidence ratings. Criteria most often not met included advance registration of the protocol, justification for excluding individual studies, risk of bias from individual studies’ being included in the review, and appropriateness of meta-analytical methods. Thus, little robust evidence is available on whether NSPT improves systemic disease outcomes [46].

Our search yielded five position papers from the Canadian Dental Hygienists Association by Lavigne and colleagues. Each was an umbrella review of systematic reviews of the evidence of a causal relationship between PD or periodontal microbes and health outcomes. Hill’s criteria for causality in a biological system were used to evaluate the evidence [47] (Table 1). At a minimum, the set of criteria includes consistency, strength of association, dose response, plausibility, and temporality. Criteria considered essential [48] are in bold font.

A 2020 review funded by the Canadian Dental Hygienists Association examined the evidence of a causal relationship between PD and CVD [49]. A total of 53 systematic reviews/meta-analyses (SRs/MAs) were retrieved, of which 7 were used in the analysis. Only one used a direct measure of CV outcome for NSPT [50]. It was judged to be of low quality, with no significant difference between treatment and control, a high risk of bias due to protocol deviation, and a lack of follow-up. The other 6 reviews were considered inadequate owing to such issues as considering only surrogate outcomes such as arterial stiffness, C-reactive protein (CRP), endothelial function, and hemoglobin A1c (HbA1c), or not finding statistically significant results from NSPT. Hill’s criteria for causality were used in the evaluation, with only biological plausibility and coherence met.

For type 2 diabetes mellitus (T2DM), 54 records were retrieved, of which 8 studies (5 SRs/MAs and 3 umbrella reviews) were reviewed [51]. All 5 SRs/MAs reported reductions in HbA1c levels 3 months after NSPT, but effect sizes were small and 2 were not statistically significant. The three umbrella reviews consistently reported small reductions in HbA1c but high levels of inconsistency and a moderate to high risk of bias. For Hill’s criteria, only plausibility and coherence were fully met, whereas temporality and experiment were partially met.

For adverse pregnancy outcomes, 37 records were retrieved, of which 9 met the criteria for inclusion and were analyzed [52]. None showed that NSPT lowered the risk of adverse pregnancy outcomes. Table 6 in that review lists 10 issues regarding systematic reviews of RCTs identified by the reviewers, including quality of studies, publication bias, evidence that does not support scaling and root planning for reducing the rate of preterm birth, selection criteria for participants, and other conditions such as smoking that were either not reported or not evaluated. For Hill’s criteria, only temporality (in a few studies) and plausibility were met. 

For periodontal microbes and respiratory diseases, the question addressed was, “For patients in hospitals, nursing homes, or long-term care facilities who are at high risk for respiratory infections, will an oral care intervention such as toothbrushing, administration of antimicrobial agents, and/or professional care, as compared to no oral care intervention (or usual oral care), reduce the risk for respiratory infections?” [53]. Of the 47 respiratory studies retrieved, after elimination of duplicates and studies not meeting inclusion criteria, 10 SRs were selected, 9 of which included MAs. Although evidence existed that locally administered chlorhexidine gluconate reduced the risk of ventilator-associated pneumonia, no effect was found for hospital-acquired pneumonia. Limitations included inconsistencies among studies in population groups, chlorhexidine gluconate concentration, frequency of administration, number of applications, and insufficient evidence for the use of povidone iodine or toothbrushing in ventilated patients. Although some studies reported other patient-centered outcomes (i.e., ICU mortality, length of ICU stay, or duration of mechanical ventilation), findings were positive only for cardiac surgery ventilated patients who did not meet the inclusion criteria. For Hill’s criteria, only plausibility and coherence were met. 

For systemic diseases, we reviewed SRs and prospective studies published between 2015 and 2021 that focused on associations between periodontitis and rheumatoid arthritis, Alzheimer’s disease/cognitive impairment, obesity, inflammatory cancers, and periodontitis and chronic kidney disease [54]. A total of 39 papers were selected for discussion, including 6 SRs for rheumatoid arthritis; 7 SRs for Alzheimer’s disease/cognitive impairment; 11 SRs, 1 metareview of SRs, and 1 population-based cohort study for obesity; 9 SRs for inflammatory cancers; and 4 SRs for kidney disease. The only one considered strong was obesity. 

An evidence brief was prepared in 2021 for the Department of Veterans Affairs, Veterans Health Administration, and Health Services Research and Development Service regarding how detecting and treating dental problems affected chronic disease outcomes [55]. The report concluded that among people with chronic obstructive pulmonary disease (COPD), periodontal treatment may improve lung function and reduce exacerbations at 1–2 years, as well as reduce annual medical costs. Among people with diabetes or CVD, periodontal treatment probably improves some measures of chronic disease severity and inflammation at 3–4 months, but benefits do not seem to persist beyond 6 months. Results were unclear on the relationship between periodontal treatment and chronic disease outcomes for people with CVD. Results were also unclear on the relation between periodontal treatment and medical costs as well as the risk of chronic disease complications among patients with diabetes, CVD, or cerebrovascular disease.

The European Federation of Periodontology held three workshops regarding PD and other adverse health outcomes. The first workshop, held in 2017 in conjunction with the International Diabetes Federation, produced a consensus report on PD and diabetes [18]. The second workshop, held in Madrid on 18–19 February 2019 in conjunction with the World Heart Federation, produced a consensus report on periodontitis and CVD [56]. The third workshop, held in Madrid in July 2022 in conjunction with the World Organization of Family Doctors, produced a consensus report regarding PD and CVD, diabetes, and respiratory diseases [57]. The final consensus report concluded that periodontitis is independently associated with CVD, diabetes, COPD, obstructive sleep apnea, and COVID-19 complications [57]. The report also recommended closer collaboration between oral health professionals and family doctors in the early detection and management of noncommunicable diseases and associated risk factors. However, the consensus reports discussed no risk factors for noncommunicable diseases other than PD. As just outlined, a review of the evidence does not support a direct role of PD in the risk of other health outcomes. However, the review can signal that risk factors affecting PD can also affect the risk of other health outcomes. Thus, we support the suggestion for closer collaboration between oral health professionals and family doctors.

As outlined in the most recent consensus report [57], the mechanisms driving adverse periodontal outcomes arising in diabetes patients with hyperglycemia are believed to include exaggerated systemic inflammation due to raised glucose levels [58]; frequency of glucose intake on systemic inflammation [59]; negative effects on neutrophil functional efficiency [60]; T-helper 1, −2, and −17 cell responses [61]; and advanced glycation end-product formation, inhibiting periodontal wound healing [62]. Thus, the evidence that diabetes is a risk factor for PD has strong support from both epidemiological and mechanistic studies.

Therefore, the saying that correlation does not imply causation is appropriate for studies reporting associations between PD and chronic or infectious diseases, meaning that the reported associations are due largely to shared underlying risk factors. The hypothesis discussed here is that dietary factors and vitamin D status are two of those underlying risk-modifying factors. The next sections explore those hypotheses. 

## 3. Dietary Risk Factors for Periodontitis/Periodontal Disease and Other Adverse Health Outcomes

Dietary factors play an important role in the etiology and risk of periodontitis, as indicated by T2DM’s strong association with periodontitis [63,64,65,66]. Dietary risk-modifying factors for T2DM include those that affect systemic inflammation and hyperinsulinemia/insulin resistance [22]. Red meat, processed meat, and sugar-sweetened drinks are risk factors, whereas whole grains, fruits, and dairy foods are risk-reduction factors [22,67,68]. 

The understanding of which dietary factors affect the risk of PD has changed. Reports regarding diet and the risk of periodontitis are discussed here chronologically. In 2000, dietary calcium was known to reduce the risk of PD [69]. By 2005, saturated fatty acids (SFAs) and nonmilk sugars were thought to increase risk, whereas diets with polyunsaturated fatty acids (PUFAs), fiber, and vitamins A, C, and E reduced risk [70]. In 2006, whole-grain consumption reduced the risk of periodontitis in a longitudinal study of US male health professionals [71]. A 2006 cross-sectional study from data in the Third NHANES reported a 20% reduced risk of periodontitis prevalence for the highest quintile of dairy intake [72]. A 2009 review reported that omega-3 fatty acids, vitamin C, lactic acid foods (dairy), soy products, and a diet rich in vegetables and fresh food appear favorable for better periodontal health, whereas a lipid-rich diet may be detrimental to periodontal tissues [73]. A 2009 report of a longitudinal study in Japan negatively correlated intake of vegetables and positively correlated intake of cereals, nuts and seeds, sugar and sweeteners, and confectioneries with PD events [74]. A 2010 longitudinal study from Japan inversely correlated consumption of docosahexaenoic acid with PD events (incidence rate ratio [IRR] for lowest vs. highest tertile, 1.49 [95% confidence interval (CI), 1.01–2.21]) [75].

A 2013 study in Germany reported that vegetarians had better periodontal conditions than nonvegetarians [76]. The cross-sectional study included 100 vegetarians and 100 non-vegetarians, with a mean age of 42 years. The vegetarians included 89 lacto-ovo vegetarians and 11 vegans. Vegetarians had significantly lower pocket-probing depths (PPDs; 2.0 ± 0.5 mm vs. 2.3 ± 0.8 mm; *p* = 0.04) and significantly lower bleeding on probing (BOP) (12 ± 13% vs. 19 ± 17%; *p* = 0.001) than nonvegetarians. The gingival recession and CAL were not significantly different between the groups. Periodontal screening indices were significantly lower in the vegetarian group (1.9 ± 1.1 vs. 2.3 ± 1.1; *p* = 0.01). The degree of tooth mobility was significantly lower in the vegetarian group (*p* = 0.04 and 0.01, respectively).

Included among the articles associating periodontitis with insulin resistance is one from Korea involving non-abdominally obese adults [77]. The study involved 29,235 participants, of whom 5690 were older than 30 years and had PD with a community periodontal index of 3 or 4. The odds ratios (ORs) for prevalence of severe periodontitis were significantly increased from normal glucose tolerance and impaired fasting glucose (OR = 1.32 [95% CI, 1.06–1.64]) to T2DM (OR = 1.5 [95% CI, 1.11–2.02]), after adjustment for potential confounders (*p*_trend_ = 0.003). The prevalence of severe periodontitis also increased significantly with increasing insulin resistance (*p*_trend_ = 0.04) in nondiabetics. Furthermore, insulin-resistant people with a healthy waist circumference showed significantly higher odds of severe periodontitis (OR = 1.47 [95% CI, 1.16–1.87]) than did insulin-sensitive people with a healthy waist circumference.

A cross-sectional study regarding diet quality and risk of severe periodontitis was conducted with 13,920 Hispanic/Latino people aged 18–74 years in the US from 2008 to 2011 [78]. The Alternative Healthy Eating Index (AHEI-2010) was used to compare dietary intakes for participants. The AHEI-2010 emphasizes high intakes of whole grains, PUFAs, nuts, and fish, as well as reductions in red and processed meats, refined grains, and sugar-sweetened beverages [79]. The study reported that the adjusted OR (aOR) for the highest versus lowest quartiles for the AHDI-2010 dietary pattern was 0.46 (95% CI, 0.30–0.72).

A study conducted in Iran looked at how a raw vegan diet affected periodontal and dental variables [80]. A raw vegan diet includes only plant foods not heated above ~118 °F. The cohort included 59 raw vegans and 59 controls of mean age 47 ± 14 years; all were nonsmokers. The comparative values for vegans and controls were as follows: PPD (mm), 1.9 ± 0.3 and 2.0 ± 0.5, *p* = 0.047; CAL (mm), 0.6 ± 0.8 and 0.6 ± 0.9, *p* = 0.98; BOP (%), 39 ± 35 and 55 ± 36, *p* = 0.02; and gingival recession (mm), 0.3 ± 0.6 and 0.3 ± 0.6, *p* = 0.85.

In Finland, a prospective study of PD development with respect to diet in 240 periodontally healthy people was conducted [81]. Participants completed food frequency questionnaires and dental exams in 2000 and 2011. Two dietary patterns were considered: the Baltic Sea Diet Score (BSDS) and the Recommended Finnish Diet Score (RFDS). The BSDS is based on a Nordic food culture and includes only healthy foods produced in Nordic countries. Positive factors were fruits, vegetables (excluding potatoes), cereals, low-fat milk, fish, and the ratio of PUFA to SFA. Negative factors were red and processed meat, the percentage of energy from fat, and alcohol. The RFDS is based on Finnish dietary recommendations but also includes foods from outside Nordic countries. It is similar to the BSDS but includes legumes and considers the ratio of white meat (poultry and fish) to red meat. Negative factors were salt, sucrose, and alcohol. The development of PD was based on the number of teeth with deepened pockets (>4 mm) in 2011. The average number of such teeth in 2011 for the BSDS was 3.2 for the lowest tertiles, 3.4 for the middle tertile, and 2.5 for the highest tertile. The respective numbers for the tertiles of the RFDS were 3.3, 3.3, and 2.4. For all participants, the adjusted continuous IRR for the BSDS was 0.95 (95% CI, 0.93–0.98), whereas for the RFDS it was 0.96 (95% CI, 0.92–0.99). For the 193 nonsmokers, the adjusted continuous IRR for the BSDS was 0.90 (95% CI, 0.87–0.93), whereas for the RFDS, it was 0.78 (95% CI, 0.74–0.81). Those results support other findings that smoking is an important risk factor for PD as well as that nonlocal foods are important for reducing the risk of PD in Nordic countries.

The Western dietary pattern has the highest direct correlation with periodontitis. A prospective study from the Harvard Health Professionals Follow-up Study was used to determine dietary links to periodontitis (reported in 2020) [82]. It involved 34,940 men free of periodontitis and other major illnesses at baseline and followed them up for 24 years. The major finding was that for those with a body mass index (BMI) >30 kg/m^2^ of body surface area, the adjusted hazard ratio (aHR) for incidence of periodontitis for the high versus low quintile was 1.83 (95% CI, 1.21–2.76). In the analysis of food groups in terms of Western diet scores, food groups with much higher servings/day for the fifth versus the first quintile were processed meat (0.81 ± 0.63 vs. 0.08 ± 0.11), red meat (1.12 ± 0.54 vs. 0.21 ± 0.16), high-fat dairy (1.57 ± 1.44 vs. 0.48 ± 0.47), eggs (0.58 ± 0.62 vs. 0.15 ± 0.19), and refined grains (1.96 ± 1.43 vs. 0.72 ± 0.60). No *p* values were given for those relationships. In the discussion, the authors noted that the Western diet is directly associated with inflammatory biomarkers [83], obesity is strongly associated with the risk of diabetes [84], and the Western diet is strongly and directly associated with insulin resistance [85].

A 2021 review discussed nutrition’s role as a key modifiable factor for periodontitis and systemic diseases [86]. The recommended diet was one low in sugar and SFAs but rich in polyols, fibers, PUFAs, vitamin A, vitamin B, vitamin C, calcium, and polyphenols.

At least two later articles reported associations between periodontitis and inflammation. One article, based on the dietary inflammatory index (DII), [87] assessed data from three NHANES surveys [88]. The DII mediated 52% of the association of mean CAL with serum CRP. A 2023 study from Korea [89] reported on 168,378 participants in a cross-sectional study and 160,397 in a prospective analysis. The energy-adjusted DII (more proinflammatory) was significantly associated with incident periodontitis (HR_Q4 vs. Q1_ = 1.29 [95% CI, 1.13–1.48]) as well as prevalent periodontitis (HR_Q4 vs. Q1_ = 1.17 [95% CI, 1.03–1.34]) for all participants. 

An investigation of an inflammatory dietary pattern and the incidence of periodontitis was reported in 2021 [90]. The study was based on 34,940 men from the Harvard Health Professionals Follow-up Study who were free of PD and major illnesses at enrollment in 1986. The men supplied medical and dental information every 2 years and completed a food frequency questionnaire every 4 years over a 24-year period. The dietary pattern was one developed at Harvard called a reversed empirical dietary inflammatory pattern (rEDIP). The analysis used nine pro-inflammatory and nine anti-inflammatory food groups. Those with the greatest change between the low and high quintiles are given in Table 1 of that article. The only significant finding was for obese nonsmokers comparing the highest quintile (the most proinflammatory diet) with the lowest quintile (HR = 1.39 [95% CI, 0.98–1.96, *p*_trend_ = 0.03]). Table 1 in that article shows that the servings/day of the key proinflammatory food groups are higher than with an optimal anti-inflammatory diet, such as red and processed meat.

A systematic review and meta-analysis of healthy dietary patterns based on 4 RCTs and 7 case-control studies was published in 2022 [91]. A clinically significant reduction was evident in BOP, gingival index, and periodontal inflamed surface area; calculus and debris index; and incidence of tooth loss in the healthy dietary patterns group, with a very low to moderate certainty of the evidence. More than half of the data were for dietary changes to the Mediterranean or vegan/vegetarian diet with a 4- to 6-week follow-up, often with significant or marginally nonsignificant improvements in PD parameters.

A 2023 article examined the association between the quality of plant-based diets and periodontitis in the general US population [92]. The study included 5651 participants from NHANES III data from 1988 through 1994, of whom 2841 had moderate-to-severe periodontitis. Adherence to a healthy plant-based diet had an OR of 0.93 (95% CI, 0.86–0.995) for periodontitis, whereas that for an unhealthful plant-based diet was 1.1 (95% CI, 1.04–1.16).

A major advancement in the understanding of dietary factors related to the risk of chronic diseases was achieved by studying three US cohorts involving 205,852 health care professionals monitored for up to 32 years [22]. Participants completed food frequency questionnaires every few years. Health outcomes were recorded and risks evaluated for 7 a priori dietary patterns and 37 dietary components. After the initial analyses, two additional dietary patterns were developed: one was a reversed empirical dietary index for hyperinsulinemia (rEDIH), which would result in a reduced risk of progression to insulin resistance; the other was rEDIP. For T2DM incidence, the aOR_90th percentile vs. 10th percentile_, including many factors (but not BMI) for rEDIH was 0.36 (95% CI, 0.35–0.37), whereas the aOR for rEDIP was 0.38 (95% CI, 0.37–0.40). With BMI, the ORs changed to 0.57 (95% CI, 0.54–0.59) and 0.57 (95% CI, 0.55–0.59). By comparison, the diabetes risk reduction diet, which evaluated the effects of sugar-sweetened beverages, coffee, nuts, red and processed meats, glycemic load, cereal fiber, PUFA-to-SFA ratio, and trans fat [93], had an aOR of 0.58 (95% CI, 0.56–0.60), and with BMI added, 0.66 (95% CI, 0.63–0.69).

The dietary factors with high risk (*r* for 90th vs. 10th percentile ≤−0.20) for rEDIH in decreasing order include red meat, french fries, processed meats, and low- and high-energy drinks. Factors for rEDIP include high-energy drinks, red meat, refined grains, and processed meats. The dietary factors protective (*r* for 90th vs. 10th percentile ≥0.20) for rEDIH in decreasing order include fruit, coffee, wine, whole grains, and leafy green vegetables, and factors protective for rEDIP include coffee, wine, and green leafy vegetables (Figure 3 in [22]). 

At this point, we can compare results from the Harvard periodontitis study [90] and the Harvard chronic disease study (Table 2). One apparent fact is that the rEDIH dietary pattern showed red and processed meats to have a more pronounced negative effect than did the rEDIP dietary pattern. Thus, the rEDIH dietary pattern would probably have had a stronger association with periodontitis in the Harvard periodontitis study than the rEDIH dietary pattern did.

A recent review outlined how ultraprocessed foods (UPFs) generate low-grade inflammation [94]. First, the quality of UPFs is low owing to the higher content of salt, SFAs, and sugar; the lower content of fiber, micronutrients, and protein; and various nonnutritive additives. The effects of UPFs are attributed largely to their effect on the gut microbiota, with increased gut permeability and lower production of short-chain fatty acids. UPF consumption is also associated with an increased risk of obesity [95] and low-grade inflammation [96]. UPFs significantly increase triglyceride concentrations [97]. 

The PREDICT study measured postprandial inflammation after each of nine “test” meals. Postprandial lipemia more strongly predicted postprandial inflammation (measured by GlycA) than did postprandial glycemia. The large interindividual variability in postprandial inflammation, partly mediated by adiposity, highlights the potential for personalized strategies to target obesity and postprandial metabolic responses to reduce low-grade inflammation [98]. The University of North Carolina’s Global Food Research Program called UPFs a global threat to health and outlined the evidence in a 10-page report published in 2021 with 204 references [99].

We searched the journal literature for representative studies regarding associations between health outcomes and dietary risk factors. Table 3, Table 4 and Table 5 give findings on how several dietary factors affect health outcomes.

Table 4 gives findings regarding meat and the risk of several adverse health outcomes. One mechanism by which meat and other animal products increase the risk of chronic diseases is that the gut biome produces a compound called trimethylamine *N*-oxide (TMAO). An international pooled analysis of data for 32,166 adults without CVD, cancer, chronic kidney disease, or inflammatory bowel disease determined the level of circulating TMAO in relation to dietary factors [107]. Factors that significantly increased concentrations per serving or per 5% of energy were, in descending order, shellfish, total fish, animal protein, eggs, saturated fat, red meat, and monosaturated fat. Nuts and plant proteins, by contrast, significantly lowered concentrations. 

A recent study conducted in Guangzhou, China, showed enhanced TMAO associated with vascular endothelial dysfunction in 122 stage III–IV periodontitis patients compared with 81 healthy controls (*p* = 0.002 [108]. Patients presented elevated TMAO (*p* = 0.002), lower endothelial progenitor cells (*p* = 0.03), and declined brachial flow-mediated vasodilation (*p* = 0.005). A mouse model study of periodontitis was used to study the effect of reducing concentrations of TMAO by using 3,3-dimethyl-1-butanol [109]. Doing so markedly improved the severity of intestinal inflammation; the gut biome was favorably altered, and alveolar bone resorption and the degree of periodontal tissue inflammation were more severe in the periodontitis + TMAO group.

TMAO has been related to an increased risk of Alzheimer’s disease [110,111,112], CVD [113,114], T2DM [115,116], and chronic inflammatory and degenerative diseases in general [117].

**Table 4:** Findings regarding dietary meat’s effects on various health outcomes.

Disease	Variable	Population	Approach	Finding	Ref.
T2DM	Processed meat	184 countries	Comparative risk-assessment model to estimate the effect of 11 dietary factors, separately and jointly, on absolute and proportional burdens of new T2DM cases among adults globally and by age, sex, education, urbanicity, world region, and nation, in 1990 and 2018.	Attributable burden 20.3% (95% CI, 18.3–23.5%)	[68]
T2DM	Red meat	184 countries	Cross-sectional study	Attributable burden 20.1% (95% CI, 19.0–21.2%)	[68]
CHD, incident	Meat, sausages	Germany, 200 cases, 255 controls	Risk per 100 g/d	HR = 2.55 (95% CI, 1.14–5.68)	[118]
IHD incident	Meat, 50 g/day;		Meta-analysis of 12 of 13 prospective studies, *N* = 1,427,989; cases, 32,630. Studies were conducted in Asia (*n* = 3), the US (*n* = 4), Australia (*n* = 1), Europe (*n* = 4), and for one multicountry cohort in the Americas, Asia, Africa, and Europe.	HR = 1.09 (95% CI, 1.06–1.22)	[119]
IHD incident	Processed meat, 50 g/day		Meta-analysis of 10 of 13 prospective studies, *N* = 1,427,989; cases, 32,630	HR = 1.18 (95% CI, 1.12–1.25)	[119]

95% CI—95% confidence interval; CHD—coronary heart disease; HR—hazard ratio; IHD—ischemic heart disease; T2DM—type 2 diabetes mellitus.

**Table 5:** Findings regarding the effect of dietary whole grains on risk of periodontitis.

Disease	Population	Approach	Finding	Ref.
Periodontitis	Harvard Health Professionals Follow-up Study 34,160 males aged 40–75, 2197 developed periodontitis	Prospective study, 1986–1998; Q1, 0.3 s/d; Q3, 1.3 s/d; Q5, 3.4 s/d	For Q5, mRR = 0.77 (95% CI, 0.66–0.89, *p*_trend_ < 0.001	[71]

mRR—multivariate relative risk; Q1—quartile 1; s/d—servings/day.

The most efficient way to reduce the risk of T2DM or reverse it is to adopt a high-quality, low-fat vegan diet. In 1979, Anderson and Ward showed in a clinical trial that a high-carbohydrate, high-plant-fiber diet could lower insulin dose requirements in men with diabetes mellitus from 26 ± 3 to 11 ± 3 units/day in 16 days [120]. In 2006, Barnard and colleagues showed in an RCT that a low-fat vegan diet improved glycemic control and CVD risk factors in participants with T2DM [121]. By week 22, mean HbA1c had decreased from 8.1 ± 1.2% to 6.8 ± 0.8% for 40 participants in the vegan arm, compared with 7.9 ± 0.9% to 7.5 ± 1.0% in the group following American Diabetes Association guidelines. However, people on low-fat vegan diets have to be advised on ways to obtain enough calcium, vitamin B_12_, and vitamin D [122], and low-fat diets may not be best for overall health. A cohort study conducted in Korea with 42,192 participants followed up from 2007 to 2015 examined the relationship of carbohydrates, fat, and protein percentages in the diet with all-cause mortality rates [123]. Optimal percentages were 50–60% for carbohydrates, 30–50% for fat, and 15–25% for protein.

A 2009 cross-sectional study of participants in the Adventists’ Health Study showed that the type of vegetarian diet affected the prevalence of T2DM [124]. Compared with 28,761 nonvegetarians, 2731 vegans had a fully adjusted OR for T2DM of 0.51 (95% CI, 0.40–0.66) or 0.32 (95% CI, 0.25–0.41) without BMI; 20,408 lacto-ovo vegetarians had an aOR of 0.54 (95% CI, 0.49–0.60) or 0.43 (95% CI, 0.39–0.47) without BMI; 5618 pesco-vegetarians had an aOR of 0.70 (95% CI, 0.61–0.80) or 0.56 (95% CI, 0.49–0.64) without BMI; and 3386 semivegetarians had an aOR of 0.76 (95% CI, 0.65–0.90) or 0.69 (95% CI, 0.59–0.81) without BMI.

Several reviews supported the role of a high-quality plant-based diet in reducing the risk of T2DM, e.g., [125,126]. In 2020, the American College of Lifestyle Medicine published a position statement supporting a whole-food, plant-based diet for T2DM remission treatment [127]. A later review also supported plant-based eating for T2DM prevention and treatment, noting that practical considerations include education, nutrition adequacy, and adjusting medications [128].

A 2021 report detailed an analysis of the incidence of T2DM with respect to changes in plant-based diet indices in two Harvard cohorts, the Nurses’ Health Study (1986–2012) and Nurses’ Health Study II (1991–2017), involving 76,530 women, and the Health Professionals Follow-up Study, involving 34,486 men [129]. Each 10% increment in the plant-based diet index (PDI) and healthful PDI (hPDI) over 4 years was associated with a 7–9% lower risk (pooled HR for PDI, 0.93 [95% CI, 0.91–0.95]; hPDI, 0.91 [95% CI, 0.87–0.95]).

## 4. Vitamin D Status Modifies Risk of Periodontitis/Periodontal Disease and Other Adverse Health Outcomes

Vitamin D is a fat-soluble secosteroid hormone that regulates calcium, magnesium, and phosphate homeostasis and plays a pivotal role as an antiproliferative and immunomodulatory mediator [130]. Vitamin D_3_ (cholecalciferol) is produced in the human body by the action of solar ultraviolet-B (UVB) radiation (290–315 nm) on 7-dehydrocholesterol in the skin, followed by a thermal reaction. That zoosterol then travels through the blood to the liver, where it receives a hydroxyl group to become 25(OH)D, the metabolite measured to assess vitamin D status. The pleiotropic effects of vitamin D are caused by calcitriol bound to vitamin D receptors coupling to chromosomes to regulate gene expression (or through rapid nongenomic effects on intracellular calcium). As organs require calcitriol, they activate it locally from 25(OH)D in situ [131]. A 2019 study showed that the number of genes controlled by vitamin D in blood cells increased from 162 on 600 IU/d of vitamin D_3_ for 16 weeks to 320 on 4000 IU/d and to 1289 genes on 10,000 IU/d [132]. 

Although 10,000 IU/d may seem excessive, the human body can produce 10,000–25,000 IU in a few hours with whole-body UVB exposure [133]. Few adverse effects result from taking 10,000 IU/d, which can raise serum 25(OH)D concentrations to 40–100 ng/mL [134]. The main adverse effect of taking high vitamin D doses and achieving high 25(OH)D concentrations is hypercalcemia. Symptoms include neuropsychiatric manifestations such as lethargy, confusion, irritability, depression, hallucinations, and, in extreme cases, stupor and coma; gastrointestinal symptoms such as anorexia, nausea, vomiting, and constipation; cardiovascular manifestations such as ectopy and sudden death; and renal symptoms such as polyuria and renal colic from the passage of renal stones [135]. Hypercalcemia generally does not occur with serum 25(OH)D concentrations below 150 ng/mL. Most cases of hypercalcemia are the result of labeling errors or vitamin D dosing errors, such as taking 50,000 IU of vitamin D daily rather than weekly. In a striking example, a person took 1 million IU/day of vitamin D_3_ because of a manufacturing error. His serum 25(OH)D concentration reached 900 ng/mL, and he became incapacitated [136]. After diagnosis and treatment, when his 25(OH)D concentration fell below 400 ng/mL, he recovered fully.

Vitamin D has many mechanisms by which it maintains good health and reduces the risk of adverse health outcomes. Two recent reviews by Holick and colleagues serve as good overviews of vitamin D’s mechanisms for maintaining health [137,138]. Table 6 summarizes mechanisms identified for reducing the risk of several adverse health outcomes.

Because most vitamin D comes from solar UVB exposure, serum 25(OH)D concentrations vary, with peak and nadir concentrations lagging peak and nadir solar UVB doses. That effect can be seen by comparing findings from two articles showing seasonal variations in serum 25(OH)D concentrations. One study is from the UK for 45-year-olds from 2002 to 2004 [149], the other for adults in the US from 2007 to 2009 [150], with solar UVB doses varying with season and latitude, as shown in a modeling study [151]. Vitamin D cannot be produced from solar UVB for about half the year at latitudes above about 40° because vitamin D can be produced only when the solar elevation angle is greater than about 45° [152]. That fact prompts the question of why wintertime serum 25(OH)D concentrations are 50–70% of peak summertime concentrations. The main reason is that vitamin D stored as 25(OH)D in muscles is recycled into the serum [153,154]. Another reason is that vitamin D is stored in fat tissue and can be released into the serum over time. In Norway, participants in a vitamin D supplementation study were followed up for a year after the end of the study [155]. The 41 who had been supplementing with 20,000 IU of vitamin D_3_/week for 5 years had a mean serum 25(OH)D concentration of 49 ng/mL compared with 28 ng/mL for the 34 in the control group. At the end of the year, those who had been supplemented had a mean serum 25(OH)D concentration of 34 ng/mL, whereas those in the control group had a mean 25(OH)D concentration of 29 ng/mL. A further minor factor is that animal products such as meat and fish are sources of vitamin D [156]. Animal product consumption as a fraction of total dietary energy increases with latitude, in part because animal products can be stored in colder environments longer, whereas plant sources of food cannot be readily grown at high latitudes in winter.

Serum 25(OH)D concentrations generally decrease with age. The main reason is that solar UVB exposure is the most important source of vitamin D for most people, and the substrate for forming vitamin D, 7-dehydrocholesterol, decreases with age. A study on that topic was conducted in Wyoming with 30 healthy people with skin types II/III, 18 aged 20–37 years and 12 aged 51–69 years [157]. Each was exposed to the sun for 30 min near solar noon between 68 and 4 days before the solstice (June 21). The average body surface area exposed was estimated to be 0.78 ± 0.02 m^2^ in the younger cohort and 0.73 ± 0.03 m^2^ in the older cohort. Peak serum 25(OH)D concentration generally increased 24–48 h after sun exposure, and those values were used to generate a model for vitamin D production by decade of age. The model indicated that vitamin D production decreases by 13%/decade, or approximately 50%, between ages 20 and 70.

Several types of evidence causally link vitamin D to a reduced risk of periodontitis and PD. Support for vitamin D’s important role in reducing the risk of periodontitis comes from a cross-sectional study from NHANES 2009–2012 involving 7246 participants [158]. The fully adjusted ORs for periodontitis prevalence by quartile of 25(OH)D were as follows: Q1 (14 ng/mL [95% CI, 3–18 ng/mL]), 1.00; Q2 (22 ng/mL [95% CI, 18–25 ng/mL]), 0.68 (95% CI, 0.54–0.85); Q3 (29 ng/mL [95% CI, 25–33 ng/mL]), 0.64 (95% CI, 0.50–0.83); and Q4 (38 ng/mL [95% CI, 33–150 ng/mL]), 0.63 (95% CI, 0.51–0.78). According to Figure 3 in that article, the OR decreased in a quasi-linear fashion from 4 to 20 ng/mL.

Further strong support for the important role of vitamin D in reducing the risk of periodontitis comes from RCTs regarding vitamin D supplementation for PD patients. Table 7 summarizes the results of those RCTs. Results were generally positive, except for one study in India [159].

The mechanisms by which vitamin D reduces the risk of periodontitis and PD are well known. They include effects on bone and cartilage formation, reduced risk of inflammation, improved immune protection, improved endothelial integrity, and reduced production of matrix metalloproteinases (MMPs). Several recent reviews have discussed those mechanisms [165,166,167,168,169].

Vitamin D’s classic mechanism, regulating calcium and phosphorus absorption and metabolism, is crucial for good oral health. When 25(OH)D concentrations are low, rickets can develop, including rachitic teeth. A review discussed the role of vitamin D in regulating phosphate-regulating endopeptidase homolog, X-linked fibroblast growth factor 23, and dentin matrix protein 1, all important for proper systemic bone mineralization [170].

Bacteria play a major role in the development and progression of PD. A 2006 review serves as a good introduction to the topic [171]. The oral bacteria can be divided into two groups: beneficial commensal and periodontopathogenic bacteria. Beneficial commensal bacteria include various facultative gram-positive bacteria such as *Streptococcus sanguinis*, *Streptococcus mitis*, *Actinomyces naeslundii*, and *Actinomyces viscosus.* Bacteria such as *Porphyromonas gingivalis*, *Aggregatibacter actinomycetemcomitans*, *Tannerella forsythia*, *Treponema denticola*, and *Eikenella corrodens* have been associated with CP since it was known that Toll-like receptors, part of the immune system, could recognize beneficial and periodontopathogenic bacteria; the role of antimicrobial peptides such as defensins in regulating concentrations of bacteria; and the effect of bacteria on modulation of cytokines/chemokines. What was not then known was vitamin D’s role in those processes. In 2006, researchers discovered that Toll-like receptor activation of human macrophages upregulated expression of the vitamin D receptor and the vitamin D-1-hydroxylase genes, leading to the induction of the antimicrobial peptide cathelicidin and the killing of intracellular *Mycobacterium tuberculosis* [172]. Thus, vitamin D-induced production of defensins and cathelicidin can kill periodontopathogenic bacteria. In 2008, Bikle noted that by shifting from T-helper 1 (Th1) to Th2 cytokine production, vitamin D could probably reduce bone loss in PD [173]. A 2020 observational study in Italy reported significant increases in salivary cytokines and reductions in 25(OH)D concentrations with increasing stages of PD [174]. A 2021 review discussed the mechanisms involved in regulating periodontal ligament cell production of proinflammatory cytokines and the known roles of vitamin D and cathelicidin in reducing such production [167]. 

Periodontitis is associated with imbalanced immune homeostasis in the oral mucosa and increased bacterial growth and multiplication in the dental plaque [174]. Most of the microorganisms involved induce a decrease in pH and a reduction in the local redox potential, with the upregulation of inflammatory mediators [175] leading to the onset and progression of the disease [176]. Immune responses in PD are imbalanced, with an increased level of proinflammatory cytokines in the saliva, including interleukin 1 (IL-1), tumor necrosis factor α (TNF-α), gamma interferon (IFN-γ), IL-17, and IL-6 [177,178]. That study also reported increased concentrations of MMP-9 in the saliva, where cytokine levels directly correlated with the severity of PD and inversely correlated with 25(OH)D concentrations. 

MMPs’ role in the pathogenesis of PD is also important. MMPs are collagenases, which are important for PD because type 1 collagen is the major component of the periodontal extracellular matrix [179]. A 2002 study showed that a year’s vitamin D supplementation in 41 healthy British Bangladeshi adults reduced plasma MMP-9 concentrations by 68% (*p* < 0.05) and CRP by 23% (*p* < 0.05) and that serum 25(OH)D concentration was the sole determinant of circulating MMP-9 [180]. A 2020 study reported that salivary MMP-8 concentration was a more robust indicator of PD stage and grade than was BOP [181]. Luchian and colleagues [179] reported that one collagenase (MMP-8) and one gelatinase (MMP-9) were also important diagnostic tools for guiding treatment of periodontitis.

Yet another way to show that vitamin D reduces the risk of periodontitis is by examining observational studies in which serum 25(OH)D concentrations are compared between periodontitis patients and matched controls. Table 8 gives data from studies that could be used. Two studies, highlighted in gray, were not used because cases and controls were not carefully matched in the large datasets. The standard deviation between mean 25(OH)D concentrations for chronic PD patients and controls was calculated as the square root of the sum of the squares of the standard deviation for each mean 25(OH)D concentration. As shown in Figure 1, using serum 25(OH)D concentration as an indication of PD is better for population mean serum 25(OH)D concentrations above ~20 ng/mL. Serum 25(OH)D concentration varies seasonally with solar UVB doses, with a lag of about 6 weeks. In the US for 2007–2009, the peak mean 25(OH)D concentration was 27–29 ng/mL in August, whereas the nadir of the mean 25(OH)D concentration was 20–22 ng/mL in February [150]. In the UK in 2002–2004, the peak mean 25(OH)D concentration for men aged 45 years was near 30 ng/mL in September, whereas the nadir for mean 25(OH)D concentration was near 14 ng/mL in February or March [149]. Thus, the season has to be considered when using the results in Figure 1 as a guide.

Evidence that vitamin D reduces the risk of other adverse health outcomes is available from RCTs, observational studies in which some participants took vitamin D supplements, and Mendelian randomization (MR) studies. Although the medical system prefers RCTs for evidence, that approach is problematic for vitamin D because vitamin D RCTs are designed mostly according to guidelines for pharmaceutical drugs. The basic assumption for such trials is that the trial is the only source of the drug. That is not the case for vitamin D because most people have access to external sources, such as solar or artificial UVB exposure; animal products, including meat, fish, eggs, and fortified milk [156]; and supplements. Heaney outlined guidelines for clinical trials of nutrients in 2014 [192]. The important aspects of vitamin D include:

- Measuring serum 25(OH)D concentrations for all prospective participants;
- Enrolling those with concentrations associated with increased risk for the expected outcome;
- Supplementing with vitamin D doses high enough to raise serum 25(OH)D concentration to maximize the effect of vitamin D;
- Normalizing all participants for potentially outcome-affecting variables not directly related to vitamin D, such as diet and vitamin A intake; and
- Measuring and analyzing results with respect to achieved 25(OH)D concentrations.

However, few vitamin D RCTs have followed those guidelines, as noted in two reviews in 2022 [193,194]. 

Regarding CVD, when the VITAL study was designed around 2010 [195], the thinking was that the risk was significantly greater for people with serum 25(OH)D concentrations <15 ng/mL on the basis of an observational study in Utah [196]. That value has been supported in an observational study in China [197] and an MR study based on data from the UK Biobank [198]. That value is also consistent with the fact that CVD events and deaths are about 25% higher in winter [199,200], when serum 25(OH)D concentrations are much lower than in summer [149,150]. Thus, with the mean baseline 25(OH)D concentration for people in the vitamin D treatment arm in the VITAL study who provided values averaging ~31 ng/mL, not enough had a 25(OH)D concentration <15 ng/mL to correctly assess the value of vitamin D supplementation. 

Thus, the absence of evidence from RCTs for particular health outcomes does not mean that vitamin D has no significant benefits for those outcomes. The pharmaceutical industry sees the promotion of vitamin D as a serious threat to its income and profit. Drug companies thus use the disinformation playbook to discredit vitamin D and restrict discussion of it in top medical journals, the mass media, medical universities, and medical systems [201]. The fact that most vitamin D RCTs reported to date found no significant health benefits may be one result of that approach.

Table 9 presents evidence from RCTs that vitamin D reduces the risks of other adverse health outcomes, including asthma, autoimmune disease, cancer, dental caries, osteoporosis, and respiratory tract infections. The number of such RCTs has been limited by poor design, conduct, and analysis because of the problems discussed above, including basing the trials on guidelines for pharmaceutical drugs rather than nutrients, as also discussed in two recent reviews [193,194].

Table 10 presents results from selected open-label vitamin D supplementation studies and observational studies based on data from vitamin D RCTs. That type of study yields important information because solar UVB exposure, the most important source of vitamin D for most people, can have non-vitamin D health benefits such as liberating nitric oxide from nitrate stores in the skin. For example, increases in serum nitric oxide from solar UV radiation lower blood pressure [209] and reduce the risk of COVID-19 [210]. As long as the controls are well matched to the cases, such results should be comparable to those of well-designed RCTs. Propensity score matching is one way to match cases and controls [211].

With the identification of genetic variants associated with variation in serum 25(OH)D concentrations, MR can now be used to examine evidence for the causal effect of vitamin D both on vitamin D status and on many health problems [221]. The method is sometimes called “nature’s controlled trial” because people are automatically randomized into study groups by the genetic variants they carry (owing to the random allocation of genetic variants during the formation of gametes). That approach reduces errors due to confounding and reverse causation, which are likely to affect other types of observational studies [222]. 

Genome-wide association studies (GWAS) of single-nucleotide polymorphisms (SNPs) for alleles of genes in the vitamin D pathway for people of comparable ethnicity within large databases can be used to randomize participants, which reduces the effect of vitamin D sources in the vitamin D pathway. The vitamin D pathway has many genes, yet most MR studies have used only a few of them because the MR approach is still being developed. Several MR studies have reported no beneficial effects related to genetically determined variation in 25(OH)D concentrations, sometimes because of having too few participants (the best studies often include more than 100,000 participants), and sometimes because the number of 25(OH)D concentrations measured was too low. In 2018, MR studies were considered able to reveal proof of causality whose validity lies between that of RCTs and observational studies [223]. With the inclusion of more SNPs, larger databases, and stratified baseline serum 25(OH)D concentrations, the ability of MR studies to determine causality is improved by using these nonlinear analyses. However, several MR studies have not shown beneficial effects of vitamin D, which could be the result of too few participants, the use of too few SNPs in analysis, or not using stratified serum 25(OH)D concentrations to show the benefit of normalized vitamin D status.

Table 11 presents results from MR studies that have reported beneficial effects related to vitamin D. MR studies support the role of vitamin D in reducing the risk of all-cause mortality, Alzheimer’s disease, CVD, and T2DM. A recent umbrella review of vitamin D and multiple health outcomes also includes hypertension, multiple sclerosis, and schizophrenia as diseases for which MR studies showed that genetically predicted 25(OH)D concentration was significantly inversely correlated with outcome. [224].

Although support for vitamin D supplementation’s reducing risk of cancer incidence and mortality rates is marginal, strong evidence is available from geographical ecological studies with respect to solar UVB doses, observational studies based on serum 25(OH)D concentrations, and studies of mechanisms whereby vitamin D reduces the risk of cancer [140]. It is also understood why vitamin D is more important for reducing cancer mortality rates than cancer incidence rates. For instance, many competing cancer risk-modifying factors operate at the cellular level, including toxins, free radicals, vitamin D, and the like. However, for angiogenesis and metastasis, vitamin D is one of the few compounds that reduces both processes.

The benefits of vitamin D for pregnancy and birth outcomes are well supported [231]. Observational studies showed reduced risks of primary cesarean delivery [232], gestational diabetes for serum 25(OH)D concentrations between 16 and 36 ng/mL [233], preterm delivery [219], and preeclampsia [234]. A trial conducted in Iran reported that vitamin D supplementation reduced the risk of gestational diabetes, preeclampsia, and preterm birth [220].

Table 12 presents representative recommendations regarding vitamin D supplementation and serum 25(OH)D concentrations for the general population. Governments generally recommend enough vitamin D supplementation to avoid vitamin D deficiency, rickets, and osteomalacia, whereas experts recommend enough supplementation to achieve 25(OH)D concentrations associated with good, if not optimal, health in studies over the past 10 years. Most countries’ recommendations are probably influenced in great measure by Big Pharma, which sees vitamin D as a threat, as discussed.

Obese people (BMI > 30 kg/m^2^) require higher vitamin D doses than others, partly because their greater mass causes a dilution effect [246]. In addition, overweight or obese people have low-grade systemic inflammation [96], a risk factor for many diseases [247]. Also, as shown in a mouse-model study, obesity decreases the activity of hepatic 25-hydroxylase (through decreased expression of *CYP2R1*), reducing conversion of vitamin D_3_ to 25(OH)D_3_ [248]. As shown in the VITAL study, even though serum 25(OH)D concentrations were raised about 12 ng/mL with 2000-IU/d vitamin D supplementation for 3 BMI categories, <25, 25–30, and >30 kg/m^2^, only people with BMI <25 had a significantly reduced risk of overall cancer incidence [195]. Unfortunately, higher serum 25(OH)D concentrations or vitamin D supplementation apparently do not reduce systemic inflammation associated with obesity in a clinically relevant manner [249]. Thus, for optimal health, overweight or obese people would do better to adopt a weight-loss diet, such as a healthy, low-inflammatory, low-hyperinsulinemic diet [22].

## 5. Discussion

This review has several strengths. It covers the recent development of an understanding of the associations between PD and other adverse health outcomes. It enables an appreciation that those associations are not causal but rather due to shared underlying risk-modifying factors. Those factors include various dietary patterns and food groups, smoking, and low vitamin D status, which increase the risk of both PD and associated adverse health outcomes, though the magnitude of the associations varies. An important limitation is that the ability to show the benefits of vitamin D supplementation to reduce the risk of PD and other adverse health outcomes is limited by the inherent difficulties in carrying out vitamin D RCTs [193,194]. Evidence from other types of studies offers strong support but is generally considered less powerful because of possible confounding by factors such as variable sun exposure and meteorological conditions, including temperature [200], as well as dubious matching of cases to controls. The lack of study data for dietary patterns, food groups, and serum 25(OH)D concentrations in studies reporting associations between PD and other adverse health outcomes was another major limitation.

This review does, however, outline evidence that several specific lifestyle factors significantly affect the risk of PD as well as the many associated health problems. Changing those lifestyle factors at the population or individual level presents many hurdles. For example, obesity is strongly linked to lower education achievement [250], ethnicity/race [250,251], and lower income level [250], notably in the southern US [44], and management of obesity is challenging [252]. Also, though smoking rates are falling, ~20% of adults were still smokers in 2021 [253].

Changing dietary habits and reducing consumption of unhealthful food groups is increasingly hard because of economic inflation and the role of corporations in producing and marketing profitable foods that are not necessarily good for health. The US government’s dietary guidelines include protein foods, including lean meats, poultry, and eggs; seafood; beans, peas, and lentils; and nuts, seeds, and soy products [254]. In 2019, the American Diabetes Association posted a report on dietary therapy for adults with diabetes or prediabetes [255]. The report offers guidance on eating patterns and their potential benefits, such as reduced diabetes risks with lower red meat consumption and with types of low-carbohydrate diets that reduce HbA1c, weight, and blood pressure, though not the risk of diabetes. A sidebar in that report gives consensus recommendations for eating patterns that emphasize reducing overall carbohydrate intake, minimizing sugar and refined grain intakes, and choosing whole foods over highly processed foods as far as possible, but without mentioning red meat or other animal products. The American Diabetes Association website lists seven major pharmaceutical or medical corporations as “Pathway to Stop Diabetes” corporate sponsors, though those organizations have little incentive to reduce the prevalence of diabetes. Also, the meat industry aims to promote and protect its product sales. For example, The Guardian recently reported how the US beef industry is creating an army of influencers and citizen activists to help promote its aims and reduce concerns about the industry’s environmental effects [256].

It would be helpful for oral health professionals to review this report. If they agree with it, they could promote the concept that PD indicates poor health and increases the risk of more serious adverse health outcomes as people age. Therefore, taking steps to change what aspects of their lifestyle they can—especially regarding dietary patterns, food choices, smoking, and serum 25(OH)D concentrations—would be useful. Those steps ought to be an integral part of the service provided by oral health professionals. We also support the suggestion for closer collaboration between oral health professionals and family doctors. 

## 6. Summary and Conclusions

For nearly a century, researchers have known that people who develop PD are at increased risk of developing many other types of adverse health outcomes. Until about 2020, it was generally thought that the associations between PD and such outcomes might be caused by systemic inflammation or by the effect of periodontopathic bacteria. However, it is now realized that the associations are not causal but due largely to shared risk factors such as dietary pattern/food groups, obesity, smoking, and low vitamin D status. Since PD generally occurs at a younger age than the associated diseases or adverse pregnancy outcomes, PD could be regarded as a signal that some lifestyle changes would help reduce the risk of further adverse health outcomes. Because oral health professionals are likely to be the first to identify PD, they should routinely inform patients with PD that they could reduce their risk of tooth loss—and of many other adverse health outcomes—by making lifestyle changes relevant to the identified risk factors. Raising serum 25(OH)D concentrations would be the easiest improvement to make, together with increased plant food intake. Patients of oral health professionals could start taking 2000–4000 IU/d (50–100 mcg/d) of vitamin D to raise serum 25(OH)D concentrations to the range of 30–50 ng/mL (75–125 nmol/L). Obese people should take higher doses and aim to change their diet to a more healthful one. Measuring serum 25(OH)D concentrations routinely would not be necessary because the doses suggested here are considered safe, but measuring them could be useful to determine whether adequate vitamin D status is being achieved. Dietary changes should also be advised through consultation with an appropriate health practitioner.

## Figures and Tables

**Table 1:** Hill’s criteria for causality [47,49].

Criterion	Meaning
**Strength of** **association**	OR or RR between 1 and 2 is considered weak, whereas >2.0 is considered strong.
**Consistency**	Similar findings in different situations.
Specificity	A factor influences specifically a particular outcome or population.
**Temporality**	The causal agent should precede the incidence of an expected outcome.
**Biological** **gradient**	A monotonically changing dose-response relationship.
**Plausibility**	Mechanisms exist to explain the effect.
Coherence	The interpretation should not seriously conflict with generally known facts of the natural history and biology of the disease.
Experiment	RCTs are generally the strongest type of evidence. They should be for disease outcomes, not merely biological parameters related to diseases. In the absence of RCTs, prospective studies may be used.
Analogy	Analogous exposures for demonstrated outcomes.

OR—odds ratio; RCT—randomized controlled trial; RR—relative risk. Essential criteria [48] are in boldface.

**Table 2:** Comparison of dietary factors from the Harvard periodontitis study [90] and the Harvard dietary patterns for prevention of chronic disease study [22].

Food Groups	Q1 Intake (Servings/Day) [90]	Q5 Intake (Servings/Day) [90]	rEDIH *r* [22]	rEDIP *r* [22]
**Proinflammatory**				
French fries			−0.39	−0.14
High-energy drinks	0.20 ± 0.34	0.52 ± 0.75	−0.20	−0.25
Low-energy drinks	0.33 ± 0.63	0.89 ± 1.54	−0.21	−0.17
Processed meats	0.30 ± 0.32	0.55 ± 0.64	−0.35	−0.17
Red meat	0.55 ± 0.40	0.81 ± 0.59	−0.48	−0.22
Refined grains	0.96 ± 0.79	1.80 ± 1.52	−0.04	−0.22
**Anti-inflammatory**				
Beer	0.67 ± 1.13	0.11 ± 0.26	0.08	0.18
Coffee	3.40 ± 2.14	0.94 ± 1.24	0.31	0.45
Fruit juice	0.93 ± 1.15	0.67 ± 0.70	0.11	0.09
Leafy green vegetables	0.95 ± 0.65	0.61 ± 0.51	0.20	0.29
Whole grains			0.22	0.15
Wine	0.65 ± 0.96	0.08 ± 0.17	0.27	0.22

Q1 and Q5 are the lowest and highest quartiles of the rEDIP pattern, respectively. For the data from [22], *r* is the correlation coefficient (negative values indicate proinflammatory) for the 90th vs. 10th percentile. rEDIH, reversed empirical dietary index for hyperinsulinemia; rEDIP, reversed empirical dietary inflammatory pattern.

**Table 3:** Findings for UPFs as a risk factor for various health outcomes.

Disease or Outcome	Variable	Population	Approach	Finding	Ref.
T2DM	Refined rice and wheat	184 countries	Cross-sectional study	Attributable burden 24.6% (95% CI, 22.3–27.2%)	[68]
T2DM	UPFs (Q1, <215 g/d; Q2, 215–323 g/d; Q3, >323 g/d)	Spain, 20,060 adults, 175 new cases	Prospective study, 12-yr follow-up	HR = 1.53 (95% CI, 1.06–2.22)	[100]
Periodontitis, moderate/severe	UPFs	Southern Brazil, *N* = 537, 205 with initial, moderate, and severe periodontitis; results for moderate and severe periodontitis	Prospective study	Standardized coefficient = 0.11, standard error = 0.05, *p* = 0.02	[101]
Overweight/obesity	UPFs	8451 middle-aged university graduates in Spain, followed up for a median of 8.9 yrs [102]; 11,872 civil servants in Brazil 35–74 yrs followed up for a mean of 3.9 yrs [103]	Two prospective observational studies	RR = 1.23 (95% CI, 1.11–1.36)	[104]
IHD/cerebrovascular disease mortality	UPFs (14.6% vs. <6.6%)	Italy, 22,475 men, mean age 55 ± 12 yrs, 255 deaths	Prospective study, 8.2-yr follow-up	HR = 1.52 (95% CI, 1.10–2.09)	[105]
CVD mortality	UPFs (14.6% vs. <6.6%)	Italy, 22,475 men, mean age 55 ± 12 yrs, 439 deaths	Prospective study, 8.2-yr follow-up	HR = 1.58 (95% CI, 1.23–2.03)	[105]
CVD incidence/mortality	UPFs	Three prospective observational studies	Meta-analysis	RR = 1.29 (95% CI, 1.12–1.48)	[104]
All-cause mortality rate	UPFs (14.6% vs. <6.6%)	Italy, 22,475 men, mean age 55 ± 12 yrs,	Prospective study, 8.2-yr follow-up	HR = 1.26 (95% CI, 1.09–1.46)	[105]
All-cancer incidence	UPFs (Q1, 8.5 ± 2.5%; Q2, 14.3 ± 1.4%; Q3, 19.8 ± 1.9%; Q4, 32 ± 9.8%)	France, adults, median age 42.8 yrs, 2228 cases	Prospective study, 2009–2017	For Q4 vs. Q1, HR = 1.23 (95% CI, 1.08–1.40, *p*_trend_ = 0.001)	[106]

95% CI—95% confidence interval; CVD—cardiovascular disease; HR—hazard ratio; IHD—ischemic heart disease; Q1—quartile 1; RR—relative risk; T2DM—type 2 diabetes mellitus; UPFs—ultraprocessed foods.

**Table 6:** Mechanisms by which vitamin D maintains good health and reduces the risk of adverse health outcomes.

Outcome	Mechanisms	Ref.
Autoimmune diseases	Regulates adaptive immunity so that the body does not attack its own tissues	[139]
Bone health	Regulates calcium and phosphorus absorption and metabolism	[137]
Cancer	Reduces incidence by affecting cellular differentiation, proliferation, and apoptosis; reduces mortality by reducing angiogenesis around tumors and metastasis; many other mechanisms	[140]
CVD	Vitamin D deficiency activates renin-angiotensin-aldosterone system; serum 25(OH)D upregulates nitric oxide concentrations, reduces oxidative stress and regulates inflammatory pathways	[141]
Infectious diseases	Induces production of antimicrobial peptides such as human cathelicidin; reduces risk of cytokine storm	[142,143]
Infectious diseases	Inhibits expression of proinflammatory cytokines through blocking the TNF-induced NF-κB1 signaling pathway; and initiates expression of ISGs for antiviral defense program through activating the IFN-α-induced Jak-STAT signaling pathway	[144]
Inflammation	Shifts production of cytokines from Th1 (proinflammatory) to Th2 (anti-inflammatory)	[145]
Insulin resistance	Maintains pancreatic β-cell function; increases insulin sensitivity in insulin-responsive tissue; reduces serum concentrations of parathyroid hormone; regulates renin-angiotensin-aldosterone system; exerts positive effects on hepatic lipogenesis and gluconeogenesis; reduces formation of reactive oxygen species	[146]
Muscle health	Regulates oxygen consumption rate, maintains mitochondrial function, reduces risk of muscle atrophy	[147]
Pregnancy outcomes	Most effects mediated by calcitriol, whose concentrations increase during pregnancy. Calcitriol regulates calcium absorption from the GI tract. Calcitriol produced in the placenta acts as an autocrine/paracrine regulator of immunity at the fetal-maternal interface for acceptance of the fetal allograft; calcitriol regulates estradiol and progesterone secretion in the placenta; calcitriol downregulates FLT1 and vascular endothelial growth factor gene expression, thereby reducing risk of preeclampsia. Calcitriol regulates immune function through effects on cytokine production	[148]

CVD—cardiovascular disease; FLT1—FMS-like tyrosine kinase 1; GI—gastrointestinal; IFN-α—interferon alpha; ISGs—interferon-stimulated gene; Th—T-helper cell; TNF—tumor necrosis factor.

**Table 7:** Results of vitamin D (and calcium) supplementation effects on serum 25(OH)D concentration in RCTs on PD indices.

Population	Intervention: Vitamin D Dose, Ca Dose	Baseline, Achieved 25(OH)D (ng/mL)	Outcome	Ref.
Michigan, USA, 40 patients with severe PD; all received open flap debridement surgery in one segment of the mouth	Half given teriparatide (20 µg) or placebo; all given 800 IU/d and 1000 mg from 3 days before surgery to 6 wks after	Deficient (*N* = 7): 17 ± 0; 6 wks, 26 ± 4; 6 mo, 20 ± 3 Sufficient (*N* = 13): 34 ± 3; 6 wks, 41 ± 6; 6 mo, 31 ± 2	BL to 1 yr deficient vs. sufficient: CAL gain: −0.43 vs. 0.92 mm, *p* < 0.01 PPD reduction: −0.43 vs. 1.83 mm, *p* < 0.01 RLBG: not significant For teriparatide treatment, no significant effect of vitamin D on CAL and PPD, but IDR was better for vitamin D sufficient (2.05 mm) than vitamin D deficient (0.87 mm), *p* < 0.01	[160]
India, 96 adults 18–64, various stages of gingivitis	GpA, 2000 IU/d; GpB, 1000 IU/d; GpC, 500 IU/d; GpD, 0 IU/d	GpA, 22 ± 7; 52 ± 10; GpB, 27 ± 1; 44 ± 9; GpC, 24 ± 5; 37 ± 6; GpD, 28 ± 3; 29 ± 4	BL to 3 m, GI; % reduction GpA: 2.4 ± 0.5, 62% ** GpB: 2.4 ± 0.6, 53% ** GpC: 2.2 ± 0.6, 38% ** GpD: 2.3 ± 0.6, 0	[161]
Egypt, 28 patients with CP treated with SRP	T: 10,000 IU/d, 5 d/wk, 12 wk	T: 14 ± 8; 38 ± 8 C: 17 ± 6; ?	BL to 3 m, Mean diff T, C GI: −19% * PI: −10% * PPD: −26% * CAL: −13% *	[162]
China, 360 moderate-to-severe periodontitis patients	T1: 120, 2000 IU/d T2: 120, 1000 IU/d	T1, 2000 IU/d: 25 ± 2; 58 ± 2 T, 1000 IU/d: 41 ± 2; C: 26 ± 2	AL (mm): T1, −0.3 ± 0.2; T2, −0.3 ± 0.2; C, −0.2 ± 0.1; *p* = 0.048 PPD (mm): T1, −0.1 ± 0.2; T2, −0.1 ± 0.2; C, −0.1 ± 0.1; *p* = 0.043	[163]
India, 27 healthy periodontitis patients	T: 25,000 IU/wk, 6 mo	T (*N* = 13): 17; 39 C (*N* = 14): 14; 23	FMBS, NS; FMPS, NS; PPD, NS	[159]
Russia, 110 patients with moderate generalized periodontitis, 40 controls, all given conventional periodontal treatment by day 14	Gp1 (*N* = 55): 0 Gp2 (*N* = 55): 800 IU/d; 500 mg/d, 12 mo Gp3 (*N* = 40): 0	Gp1: 15 ± 0; 17 ± 0 Gp2: 15 ± 0; 37 ± 0 Gp3: 44 ± 1; 45 ± 0	BL, 12 m MI: Gp1, 2.6 ± 0.0; 2.1 ± 0.1 * Gp2: 2.7 ± 0.1; 1.1 ± 0.0 * Gp3: 2.5 ± 0.1; 1.3 ± 0.0 * PeI: Gp1: 5.4 ± 0.1; 4.7 ± 0.1 * Gp2: 5.4 ± 0.1; 3.7 ± 0.1 * Gp3: 5.3 ± 0.1; 3.6 ± 0.1 * PMA: Gp1: 53 ± 0; 33 ± 1 * Gp2: 52 ± 1; 22 ± 0 * Gp3: 53 ± 0; 19 ± 0 * SI: Gp1: 3.1 ± 0.0; 1.8 ± 0.1 * Gp2: 3.2 ± 0.1; 1.4 ± 0.1 * Gp3: 3.0 ± 0.1; 0.9 ± 0.0 *	[164]

* *p* < 0.05; ** *p* < 0.001; BL—baseline; C—control arm; CAL—clinical attachment level loss; CP—chronic periodontitis; FMBS—full mouth bleeding score; FMPS—full mouth plaque score; Gp—group; GI—gingival index; IDR—infrabony defect resolution; MI—Muhlemann index; NS—not significant; PD—periodontal disease; PeI—periodontal index; PI—plaque index; PMA—papillary-alveolar index; PPD—pocket-probing depth; RLBG—radiographic linear bone gain; SI—Svrakoff index; SRP—scaling and root planing; T—treatment arm.

**Table 8:** Serum 25(OH)D concentrations in chronic PD patients and controls; some data from [182].

Country	Year Pub.	Sex	Population CP, *N*, Age (Mean, Range), Years	25(OH)D (ng/mL) PD	Population Controls, *N*, Age (Mean, Range), Years	25(OH)D (ng/mL) Controls	Ref.
India	2023	F, M	50, 33 ± 6	13.6 ± 4.7	50, 31 ± 6	17.2 ± 6.9	[183]
USA	2021	F, M	1437, 69	29.1 ± 12.3	625, 69 yrs	33.2 ± 12.2	[88]
Italy	2020	F, M	46, 53 ± 4	17.4 ± 5.2	43, 54 ± 5	29.9 ± 5.4	[184]
Norway (Nor)	2019	M	21, 52 ± 13	19.2 ± 6.1	23, 50 ± 13	26.6 ± 7.4	[185]
Norway (Tam)	2019	M	27	13.3 ± 5.0	21	13.9 ± 3.4	[185]
Turkey	2018	F, M	55, 36 (30–47)	16.1 ± 8.3	27, 39 (21–40)	16.9 ± 6.4	[186]
USA	2018		388	19.4 ± 1.0	9308	21.8 ± 0.5	[187]
USA (Puerto Rico)	2017	F, M	19, 48 ± 9	18.5 ± 4.6	19, 49 ± 8	24.2 ± 7.1	[188]
Turkey	2017	F, M	18	14.6 ± 6.4	18	17.6 ± 9.7	[189]
Iran	2016	F	30, 34	12.3 ± 8.4	30, 34	19.3 ± 10.1	[190]
India	2016	F, M	50, 43	16.9 ± 5.5	48, 44	22.3 ± 5.7	[191]

25(OH)D—25-hydroxyvitamin D; AP—aggressive periodontitis; CP—chronic periodontitis; Nor—Norwegian; PD—periodontal disease; Tam—Tamil.

**Table 9:** Evidence from RCTs that has shown that vitamin D reduces the risk of other adverse PD-associated health outcomes.

Disease	Population	Intervention	Finding	Ref.
Asthma	7 RCTs with 95 events for 518 participants in treatment arm, 526 controls with 133 events	Vitamin D supplementation	Reduction in rate of asthma exacerbation (RR = 0.73 [95% CI, 0.58–0.92], but only for patients with serum 25(OH)D <30 ng/mL).	[202]
Autoimmune disease	25,871 participants, USA, mean 25(OH)D in treatment group ~31 ng/mL	Half given 2000 IU/d vitamin D_3_, followed up for 5.3 years	63 participants who received vitamin D and omega-3 fatty acids (HR = 0.69 [95% CI, 0.49–0.96]), 60 who received only vitamin D (HR = 0.68 [95% CI, 0.48–0.94]). Due mainly to rheumatoid arthritis.	[203]
Cancer, all, incidence, mortality rates	25,871 participants, USA, mean 25(OH)D in treatment group ~31 ng/mL	Half given 2000 IU/d vitamin D_3_, followed up for 5.3 years	Secondary analysis of an RCT for those with BMI <25 kg/m^2^: 25(OH)D increased from 33 to 46 ng/mL, HR = 0.76 (95% CI, 0.63–0.90). For all-cancer mortality after omission of the first 2 years of data HR = 0.75 (95% CI, 0.59–0.96).	[195]
Cancer, all mortality rate	For total cancer mortality, five trials were included (1591 deaths; 3–10 years of follow-up; 22–54 ng/mL of achieved circulating 25(OH)D in intervention group)	Vitamin D supplementation	RR = 0.87 (95% CI, 0.79–0.96; *p* = 0.005)	[204]
Cancer, colorectal, survival	7 RCTs with 957 colorectal cancer patients	Vitamin D supplementation	Reduction in adverse outcomes, *N* = 815, HR = 0.70 (95% CI, 0.48–0.93); progression-free survival, *N* = 340, HR = 0.65 (95% CI, 0.36–0.94)	[205]
Dental caries	17 trials from 1926 to 1942, 660 participants, mean ages 4–12 yrs	Vitamin D_3_ supplementation, 300–1400 IU/d (one with 3050 IU/d)	Reduced risk of dental caries, pooled relative rate = 0.51 (95% CI, 0.40–0.65)	[206]
Osteoporosis Review			Vitamin D deficiency symptoms are secondary hyperparathyroidism and bone loss, leading to osteoporosis and fractures; mineralization defects, which may lead to osteomalacia in the long term; and muscle weakness, causing falls and fractures.	[207]
RTIs, acute	25 eligible RCTs (total 11,321 participants, aged 0–95 years) were identified. IPDs obtained for 10,933 (96.6%) participants.		Vitamin D supplementation reduced risk of acute RTI among all participants (aOR = 0.88 [95% CI, 0.81–0.96]). In subgroup analysis, protective effects were seen in those receiving daily or weekly vitamin D without additional bolus doses (aOR = 0.8 [95% CI, 0.72–0.91])	[208]

25(OH)D—25-hydroxyvitamin D; 95% CI—95% confidence interval; aOR—adjusted odds ratio; BMI—body mass index (kilograms per square meter of body surface area); COPD—chronic obstructive pulmonary disease; HR—hazard ratio; IPD—individual participant data; MI—myocardial infarction; MR—Mendelian randomization; POCS—polycystic ovary syndrome; RCT—randomized controlled trial; RR—relative risk; RTI—respiratory tract infection; SLE—systemic lupus erythematosus; SNPs—single-nucleotide polymorphisms; T2DM—type 2 diabetes mellitus.

**Table 10:** Open-label vitamin D supplementation studies and observational studies based on data from vitamin D RCTs.

Disease or Outcome	Population	Intervention	Finding	Ref.
Airway microbial activity	40 participants in US, aged 18–60 yrs	20 supplemented with 1000 IU/d of vitamin D_3_ in summer, 20 in winter, 20 with placebo in each season	Airway surface liquid had high antimicrobial (LL-37) activity in summer without vitamin D supplementation and in winter after vitamin D supplementation	[212]
COVID-19	All people ≥18 years old living in Barcelona-Central Catalonia (*n* = 4.6 million)	Those supplemented with cholecalciferol or calcifediol from April 2019 to February 2020 were compared with propensity score-matched untreated controls	Patients on cholecalciferol treatment achieving 25(OH)D levels ≥30 ng/mL had lower risk of SARS-CoV-2 infection, lower risk of severe COVID-19, and lower COVID-19 mortality than unsupplemented 25(OH)D-deficient patients (56/9474 [0.6%] vs. 96/7616 [1.3%]; HR = 0.66 (95% CI, 0.46–0.93), *p* = 0.02)	[213]
COVID-19	VA patients with Veterans Administration Corporate Data Warehouse electronic health records. 199,498 treated, controls	Those with at least one VA service or vitamin D_3_ prescription and at least one vitamin D lab test between 1 January 2019, and 31 December 2020	For COVID-19 incidence: HR = 0.72 (95% CI, 0.65–0.79) For COVID-19 mortality: HR = 0.77 (95% CI, 0.55–1.06)	[214]
Dementia	US, 12,388 participants National Alzheimer’s Coordinating Center dataset, followed up 1 yr	1797 had calcium-vitamin D; 1046 had cholecalciferol only; 1283 had ergocalciferol only; and 511 had at least two supplements together, mostly ergocalciferol plus calcium-vitamin D (*n* = 268) and cholecalciferol plus calcium-vitamin D (*n* = 233). Age ~72 ± 8 yrs	For incident dementia, HR = 0.60 (95% CI, 0.55–0.65) Limitation: no information on serum 25(OH)D concentration available	[215]
Prediabetes to T2DM	3 RCTs included	Tested cholecalciferol, 20,000 IU (500 μg) weekly; cholecalciferol, 4000 IU (100 μg) daily; or eldecalcitol, 0.75 μg daily, vs. matching placebos	Among participants assigned to vitamin D group who maintained an intratrial mean serum 25(OH)D level of ≥50 ng/mL compared with 20–29 ng/mL during follow-up, cholecalciferol reduced risk for diabetes by 76% (HR = 0.24 [95% CI, 0.16–0.36]), with a 3-yr absolute risk reduction of 18.1% (95% CI, 11.7–24.6%).	[216]
Hypertension		Open-label vitamin D supplementation to increase 25(OH)D concn to >40 ng/mL.	For hypertensives, this reduced systolic blood pressure by 18 mmHg and diastolic blood pressure by 12 mmHg and reduced prevalence of hypertension in 71%.	[217]
MI	Patients treated at Veterans Health Administration from 1999 to 2018		Three groups: 25(OH)D <20 ng/mL; treated with vitamin D, 25(OH)D 20–30 ng/mL; treated with vitamin D, 25(OH)D >30 ng/mL. Among the cohort of 20,025 patients, risk of MI was significantly lower for >30 ng/mL vs. 20–30 ng/mL (HR = 0.65 [95% CI, 0.49–0.85]) and <20 ng/mL (HR = 0.73 [95% CI, 0.55–0.96]).	[218]
Preterm delivery	1064 pregnant patients aged 18–45 yrs between September 2015 and December 2016 in South Carolina. 46% white, 37% African American, 11% Hispanic.	Serum 25(OH)D concn measured at first prenatal visit. Women counseled on how to achieve 25(OH)D >40 ng/mL and given bottles of 5000-IU vitamin D_3_ capsules.	Overall preterm birth rate was 13%. For preterm birth, comparing >40 ng/mL vs. <40 ng/mL, OR = 0.38 (95% CI, 0.23–0.62, *p*_trend_ = 0.0003). Result largely independent of race/ethnicity.	[219]
Pregnancy outcomes: stratified randomized field trial in Iran	Iran, 900 pregnant women at screening site; 900 at nonscreening site.	Subjects with moderate deficiency I1: 50,000 IU of oral vitamin D_3_/wk for 6 wks I2: 50,000 IU of oral vitamin D_3_/wk for 6 wks and then maintenance dose of 50,000 IU/mo of vitamin D_3_ until delivery I3: Single dose of i.m. 300,000 IU of vitamin D_3_ I4: Single dose of i.m. 300,000 IU of vitamin D_3_ and then maintenance dose of 50,000 IU/mo of vitamin D_3_ until delivery Subjects with severe deficiency I5: 50,000 IU of oral vitamin D_3_/wk for 12 wks I6: 50,000 IU of oral vitamin D_3_/wk for 12 wks and then maintenance dose of 50,000 IU/mo of vitamin D_3_ until delivery I7: i.m. 300,000 IU of vitamin D_3_; 2 doses for 6 wks I8: i.m. 300,000 IU of vitamin D_3_; 2 doses for 6 wks, followed by maintenance dose of 50,000 IU/mo of vitamin D_3_ until delivery	After supplementation, only 2% of women in nonscreening (control) site had 25(OH)D >20 ng/mL vs. 53% of women in screening site. Adverse pregnancy outcomes, including preeclampsia, gestational diabetes mellitus, and preterm delivery, were decreased by 60%, 50%, and 40%, respectively, in screening (supplementation) site.	[220]

25(OH)D—25-hydroxyvitamin D; 95% CI—95% confidence interval; GRH—GrassrootsHealth; HR—hazard ratio; I1—intervention 1; i.m.—intramuscular; IQR—inter-quartile range; IU—international units; MI—myocardial infarction; OR—odds ratio; T2DM—type 2 diabetes mellitus; VA—Veterans Administration.

**Table 11:** Evidence from MR studies showing that vitamin D reduces the risks of chronic and infectious diseases associated with PD.

Outcome	Population	Approach	Finding	Ref.
All-cause mortality rate	307,601 unrelated UK Biobank participants of White European ancestry (aged 37–73 yrs at recruitment). 18,700 deaths during 14-yr follow-up.	Genetically predicted 25(OH)D estimated using 35 confirmed variants of 25(OH)D. 100 strata of 25(OH)D used.	Association of genetically predicted 25(OH)D with all-cause mortality was L-shaped (*p*_nonlinear_ < 0.001), and risk for death decreased steeply with increasing concentrations until 20 ng/mL. Evidence for association also seen in analyses of mortality from cancer, CVD, and respiratory diseases (*p* ≤ 0.033 for all outcomes).	[225]
AD	Data from International Genomics of Alzheimer’s Project (*N* = 17,008 AD cases and 37,154 controls).	SUNLIGHT Consortium identified 4 SNPs to be genomewide significant for 25(OH)D, which described 2.44% of variance in 25(OH)D in Canadian Multicentre Osteoporosis Study	A 1-SD decrease in natural log–transformed 25(OH)D (~10 ng/mL increased AD OR = 1.25 (95% CI, 1.03–1.51; *p* = 0.02).	[226]
AD	21,982 cases and 41,944 cognitively healthy controls of European descent from four consortia, including ADGC, CHARGE, EADI, and GERAD/PERADES	6 loci including GC, NADSYN1/DHCR7, CYP2R1, CYP24A1, SEC23A, and AMDHD1 from a recent GWAS including 79,366 (all European descent)	For meta-analysis of results for each SNP OR = 0.62 (95% CI, 0.46–0.84).	[227]
AD	UK Biobank. In this GWAS dataset, a proxy phenotype for AD case-control status was assessed via self-report. Participants asked to report “Has/did your father or mother ever suffer from Alzheimer’s disease/dementia?” Excluded participants whose parents were aged <60 yrs, dead before reaching age 60 yrs, or without age information.	6 loci including GC, NADSYN1/DHCR7, CYP2R1, CYP24A1, SEC23A, and AMDHD1 from a recent GWAS including 79,366 (all European descent)	For meta-analysis of results for each SNP OR = 0.88 (95% CI, 0.73–1.06; *p* = 0.19).	[227]
CVD	Nonlinear MR analysis conducted in UK Biobank with 44,519 CVD cases and 251,269 controls. Serum 25(OH)D concn instrumented using 35 confirmed genomewide significant variants.	We constructed a weighted genetic score (vitamin D-GS) consisting of 35 SNPs to instrument serum 25(OH)D concn. 100 strata of 25(OH)D used.	L-shaped association between genetically predicted serum 25(OH)D and CVD risk (*p*_nonlinear_ = 0.007), where CVD risk initially decreased steeply with increasing concentrations and leveled off around 20 ng/mL.	[198]
Pneumonia, bacterial	116,335 randomly chosen white Danes aged 20–100 yrs from the Copenhagen City Heart Study and Copenhagen General Population Study	Plasma 25(OH)D decreasing genetic variants around CYP2R1 (rs117913124, rs12794714, and rs10741657), DHCR7 (rs7944926 and rs11234027), GEMIN2 (rs2277458), and HAL (rs3819817)	In genetic analyses, OR for bacterial pneumonia per 4-ng/mL lower plasma 25(OH)D was 1.12 (95% CI, 1.02–1.23) in Wald’s ratio, 1.12 (95% CI, 1.04–1.20) in inverse variance weighted, 1.63 (95% CI, 0.96–2.78) in MR-Egger, and 1.15 (95% CI, 1.05–1.26) in weighted median instrumental variable analysis.	[228]
T2DM	96,423 white Danes aged 20–100 yrs from 3 studies. 5037 had T2DM	Assessed effects of genetic variation in *DHCR7* (related to endogenous production) and *CYP2R1* (related to liver conversion) on plasma 25(OH)D concn	OR for T2DM for 8-ng/mL reduction in genetically determined plasma 25(OH)D concn was 1.51 (95% CI, 0.98–2.33).	[229]
T2DM	Meta-analysis of 10 MR studies involving 58,312 cases and 370,592 controls	Genetic score using two 25(OH)D synthesis SNPs (DHCR7-rs12785878 and CYP2R1-rs10741657)	For 10-ng/mL increase in genetically determined 25(OH)D OR = 0.86 (95% CI, 0.77–0.97).	[230]

25(OH)D—25-hydroxyvitamin D; 95% CI—95% confidence interval; AD—Alzheimer’s disease; ADGC—Alzheimer Disease Genetics Consortium; CHARGE—Cohorts for Heart and Aging Research in Genomic Epidemiology Consortium; CVD—cardiovascular disease; EADI—European Alzheimer’s Disease Initiative; GERAD/PERADES—Genetic and Environmental Risk in AD/Defining Genetic, Polygenic, and Environmental Risk for Alzheimer’s Disease Consortium; OR—odds ratio; SNP—single-nucleotide polymorphism; T2DM—type 2 diabetes mellitus.

**Table 12:** Important recommendations regarding vitamin D supplementation and/or serum 25(OH)D concentration.

Year	Country(ies) or Region	Organization	Target	25(OH)D Concn (ng/mL)	Vitamin D Dose (IU/d) (40 IU = 1 µg)	Ref.
2010	Western developed	Experts	General health	30–100	800	[235]
2011	USA	IOM	Bone health	>20	600–800	[236]
2011	USA	Endocrine Society	Bone health	>30	1000–2000	[237]
2013	Poland	EVIDAS	General health	30–50	Adults: 800–2000; obese adults: 1600–4000	[238]
2017	40 countries	Review	General health	>12 or >20 in most guidelines	Mostly 200–800	[239]
2018	Poland	EVIDAS	General health	>30	200–2000	[240]
2018	UAE and Gulf populations	Experts	General health	30–50	400–2000, depending on age	[241]
2018	USA	Experts	Pregnancy	>40	4000–5000	[231]
2019	Europe, Middle Eastern	ECTS	General health	>20 in most guidelines	Not given	[242]
2022	Central and Eastern Europe	Experts	General health	30–50	800–2000; higher vitamin D doses (e.g., 6000 IU/d) may be used for the first 2–4 weeks	[243]
2023	Poland	EVIDAS	General health	30–50	400–4000 depending on age	[244]
2023	KSA and UAE	Experts	General health	>30	Up to 4000	[245]

ECTS—European Calcified Tissue Society; EVIDAS—European Vitamin D Association; IOM—Institute of Medicine; KSA—Kingdom of Saudi Arabia; UAE—United Arab Emirates.

## Data Availability

Not applicable.
